# Lethal and sublethal effects of chemical and bio-insecticides on *Spodoptera frugiperda* adults: new perspectives for “attract-and-kill” control strategies

**DOI:** 10.3389/fpls.2025.1694032

**Published:** 2025-10-15

**Authors:** Nourhan A. El-Said, Nawal AbdulAziz Alfuhaid, Biju Vadakkemukadiyil Chellappan, Hossam S. El-Beltagi, Mohamed M. El-Mogy, Moataz A. M. Moustafa

**Affiliations:** ^1^ Department of Economic Entomology and Pesticides, Faculty of Agriculture, Cairo University, Giza, Egypt; ^2^ Department of Biology, College of Science and Humanities, Prince Sattam Bin Abdulziz University, Al-Kharj, Saudi Arabia; ^3^ Department of Biological Sciences, College of Science, King Faisal University, Al-Ahsa, Saudi Arabia; ^4^ Agricultural Biotechnology Department, College of Agriculture and Food Sciences, King Faisal University, Al-Ahsa, Saudi Arabia; ^5^ Department of Arid Land Agriculture, College of Agricultural and Food Sciences, King Faisal University, Al-Ahsa, Saudi Arabia

**Keywords:** *Spodoptera frugiperda*, chlorantraniliprole, ingestion bioassay, fecundity suppression, detoxification enzymes

## Abstract

*Spodoptera frugiperda* (Lepidoptera: Noctuidae), commonly known as the fall armyworm, is a highly destructive migratory insect that poses a serious risk to global agricultural production, particularly maize crop. Targeting adult stages through ingestion-based control strategies offers a promising alternative to conventional broad-spectrum insecticide applications. In the current research, the lethal and sublethal impacts of five insecticides were evaluated against adult *S. frugiperda*, with a particular focus on chlorantraniliprole. Of the tested compounds, chlorantraniliprole revealed the highest toxicity (LC_50_ = 1.29 mg/L). Sublethal exposure to chlorantraniliprole significantly reduced larval and pupal development durations, decreased pupal weights, and shortened adult longevity in the offspring, without significantly affecting larval mortality, pupation rate, or emergence rate. Enzymatic assays showed a minimal induction of detoxification enzymes, suggesting a lower likelihood of rapid resistance emergence. This underscores the promise of chlorantraniliprole as an effective, environmentally favorable agent for integration into attract-and-kill strategies aimed at the sustainable control of *S. frugiperda* infestations.

## 1 Introduction

The fall armyworm, *Spodoptera frugiperda* (J.E. Smith, 1797) (Lepidoptera: Noctuidae), is a globally significant pest with an extensive host range, especially maize crops (*Zea mays* L.), and has become the most severe lepidopteran threat to maize production systems (Tepa-Yotto et al., 2023; Moustafa et al., 2024a). Owing to its nocturnal activity and post-emergence feeding behavior, *S. frugiperda* adults present an opportunity for pest control strategies that target them directly with toxicant mixtures. Like other migratory pests, the dissemination of *S. frugiperda* is greatly influenced by the adult’s ability to reproduce and disperse (Mao et al., 2023). Currently, *S. frugiperda* is documented from more than 100 countries, including Sub-Saharan, West, and Central Africa (Goergen et al., 2016; Day et al., 2017). In addition, it moves to the Asian continent in 2018 (Sharanabasappa et al., 2018), and in 2020 it reached Australia (Qi et al., 2021). Beyond life history traits, the flight capacity of this pest is a crucial concern for management efforts, especially given its highly migratory nature (Zhang et al., 2022).

Targeting bisexual adults using food attractants is one of the most effective approaches for area-wide integrated pest management (IPM) strategies against migrating pests (Wu and Guo, 2007; Zhang et al., 2022). Using the attract-and-kill strategy (Socorro et al., 2010; Gregg et al., 2018) instead of regular field spraying significantly reduces the environmental impact of pest management (Zhang et al., 2020a, b). Currently, chemical insecticides are commonly used and have lethal and sublethal consequences when used in real-world settings. Low amounts of pesticides can change essential features related to insects, including life duration, egg production, developmental stages, and mobility (Guedes et al., 2015; Lutz et al., 2018; Guedes et al., 2022). However, the repetitive application of high concentrations of insecticides often leads to resistance development (Daisley et al., 2018; Gressel, 2018). Insects respond to biological and abiotic stresses by modifying their migration habits, how they eat, reproduction, physiology, and metabolism (Sparks et al., 2021). Over time, insects have evolved multiple resistance mechanisms, such as target site, metabolic, behavioral, and penetration resistance (Khan et al., 2020). Metabolic resistance involves the rapid removal of insecticides by detoxifying chemicals using enzymes (Mokbel et al., 2024; Moustafa et al., 2024b). Therefore, it is essential to find new insecticides that can delay insects from developing resistance (Moustafa et al., 2023). In this regard, the anthranilic diamide class, developed by DuPont (Wilmington, France), exhibits unique features (Kandil et al., 2023). Chlorantraniliprole belongs to this group, and it results in the release of extra calcium, ultimately causing paralysis and death among the pests targeted (Akhtar et al., 2022). It is considered to be safe for mammals and exhibits selective action that preserves beneficial natural enemies including *Chrysoperla externa* Hagen, *Eriopis connexa* (Germar), *Podisus nigrispinus* (Dallas), and *Orius insidiosus* (Say) (MaChado et al., 2019). It works well against several species such as lepidopteran larvae, hemipteran, and coleopteran species (Lahm et al., 2009; Akhtar et al., 2022).

Various natural enzymes in insects are closely linked to biological processes and play critical roles in detoxification, including hydrolases, esterases, acetylcholinesterase, glutathione S-transferases (GSTs), carboxylesterases, and cytochrome P450 monooxygenases (Lin et al., 2015; Naik et al., 2018; Ismail, 2020; Clark et al., 2021; Yang et al., 2021; Ahmad et al., 2022; Siddiqui et al., 2022). There is evidence that *S. frugiperda* worldwide has developed resistance to roughly 47 chemical compounds (Mota-Sanchez and Wise, 2020). These include different insecticides such as organophosphates, carbamates, benzoylureas, diamides, spinosyns, pyrethroids, and *Bacillus thuringiensis* Berliner (Bt) toxins (Gutiérrez-Moreno et al., 2019; Kulye et al., 2021).

Nevertheless, the success of chemical applications may depend on understanding the sublethal effects of insecticides. Based on our knowledge, lethal and sublethal insecticidal effects have been recorded in the larval stage of several lepidopteran pests (Kandil et al., 2020; Awad et al., 2024; Moustafa et al., 2025). Thus, in the present work, the susceptibility of *S. frugiperda* moths to five common insecticides—lambda-cyhalothrin, indoxacarb, chlorantraniliprole, chlorfenapyr, and spinetoram—was evaluated. Additionally, chlorantraniliprole’s lethal and sublethal effects on bisexual adult development and enzymatic activity were assessed to determine its suitability for integration into food-attractant-based attract-and-kill control strategies.

## 2 Methodology

### 2.1 Insect

*S. frugiperda* eggs were acquired from laboratory colonies at Cairo University’s Faculty of Agriculture. The *S. frugiperda* larvae were reared on castor leaves oil (*Ricinus communis* L.) (Moustafa et al., 2024a, 2025) under laboratory conditions for over 12 consecutive generations without any exposure to insecticides (Moustafa et al., 2022) of 25 ± 1°C and 60 ± 5% relative humidity with 16:8-h light-to-dark cycle. The pupae were sorted by gender and placed in a glass jar (16.0cm height × 7.5cm diameter) until adult moths emerged. The newly emerged adult moths were used within 24h of emergence for the laboratory experiments.

### 2.2 Insecticides and chemicals

Five insecticides were utilized as technical-grade formulations (%w/v, as indicated), including lambda-cyhalothrin (95%), indoxacarb (95%), chlorantraniliprole (98%), chlorfenapyr (97%), and spinetoram (60%). All insecticides were provided by the Agricultural Research Center (Central Agricultural Pesticides Laboratory), Egypt. All chemicals including substrates and reagents that were used in the biochemical analysis were procured from Sigma Aldrich, Darmstadt, Germany.

### 2.3 Bioassay of *S. frugiperda* moths

A total of 100 mL of stock solution of the tested insecticides was prepared in N,N-dimethyl formamide and subsequently underwent dilution to five serial concentrations (ranging from 120 to 0.5 mg a.i., as shown in **Supplementary Table S1**) by adding a 10% v/v honey solution supplemented with 0.1% Triton X-100 (Zhang et al., 2022). The treated cotton ball by each concentration was placed inside a small plastic cup (2.5cm height × 4.5cm diameter), wrapped with cotton gauze (having a gap of 0.7mm) to minimize direct contact of the adult to the insecticide (Zhang et al., 2020), and put inside a glass jar (13.5cm height and 7.5cm diameter) with five *S. frugiperda* moths (24 days old) that were randomly chosen. It was exposed as one replicate (five replicates for each concentration). After 24h from exposure, the mortality was recorded to calculate the LC values (the moths have lost their ability to fly) (Wu and Guo, 2005).

### 2.4 Lethal and sublethal effects on reproduction

Five *S. frugiperda* moths (same sex) were transferred to a glass jar as described above (Section 2.3) and provided with a cotton ball saturated with sublethal and lethal concentrations (LC_10_ and LC_50_) of chlorantraniliprole or with 10% honey solution supplemented with 0.1% Triton X-100 for the control. A total of 40 replicates (200 female and 200 male moths) were used for the LC_50_ treatment, 20 replicates (100 female and 100 male) for the LC_10_ treatment, and 30 replicates (150 female and 150 male) for the control. After 24h from exposure, the surviving female and male moths from both treatments and control were paired. The pairing was done in the following order: LC_10_♀ + LC_10_ ♂, LC_10_♀ + CK♂, CK♀ + LC_10_♂; LC_50_♀ + LC_50_♂, LC_50_♀ + CK♂, CK♀ + LC_50_♂; CK♀ + CK♂. Each pair was transferred to a glass gar (11.5cm height × 5.5cm diameter) and fed with 10% honey solution only. Within each jar, the cotton ball was changed every day, and the number of eggs and egg hatching, respectively, were recorded. Three replicates (10 pairs/replicates) were performed for each treatment.

### 2.5 Lethal and sublethal effects on traits of offspring

Freshly hatched *S. frugiperda* offspring larvae were randomly selected from each treatment and placed into a plastic cup (9.0cm diameter × 5.5cm height) (one larvae per cup) with untreated castor leaves to assess chlorantraniliprole’s sublethal and lethal impacts on *S. frugiperda* growth. Three replicates (30 larvae per replicate) were performed for each treatment and kept under laboratory conditions (Moustafa et al., 2023). The insects were examined, and the castor leaves were changed daily. The development parameters were recorded using several key parameters, namely: larval and pupal durations, pupation (%), pupal weight, adult emergency (%), and female and male ratio (%).

### 2.6 Assay of enzymatic activity

#### 2.6.1 Preparation of samples

Surviving adults (male and female) from the LC_10_, LC_50_, and control treatments were utilized in the enzyme activity assays (Zhang et al., 2020). After treatment with LC_10_ and LC_50_ of chlorantraniliprole, three of the individual adults (~250 mg) per sex were homogenized after removing the wings in 2 mL of 0.1 M phosphate buffer, pH 7. Then, the homogenates underwent 15-min centrifugation at 12,000 *g* and 4°C, and the supernatants were taken in new tubes as a source of enzyme. All treatments were performed three times per sex. The method of Bradford (1976) was employed to assess the total protein.

#### 2.6.2 Carboxylesterases activity

Carboxylesterases (CarE) activity was performed following Van Asperen (1962) by using α-naphthyl acetate (α-NA) as the substrate. Then, 100 µL of α-NA (30mM) was incubated with 30 µL of enzyme source for 15min at 30°C. Next, 50 µL of a solution composed of a 2:5 mixture of 1% Fast Blue B and 5% sodium dodecyl sulfate (SDS) was dispensed into each sample. The optical density (OD) value was recorded at 600 nm, utilizing a Jenway Spectrophotometer-7205 UV/Vis., UK, considering α-naphthol as a reference.

#### 2.6.3 Cytochrome P-450 activity

The activity of cytochrome P-450 was tested following the technique outlined in Hansen and Hodgson (1971). Then, 100 µL of 2 mM p-nitroanisole was incubated with 90 µL of enzyme solution for 2min at 27°C. Next, 9.6 mM of NADPH was added, and p-nitrophenol was considered as a standard. The OD value was recorded at 405 nm by using a microplate reader (Clindiag-MR-96, Steenberg, Belgium).

#### 2.6.4 Glutathione S-transferase activity

Glutathione S-transferase (GST) activity was determined following Habig et al. (1974). Supernatant at 10 µL of was blended with 30 mM of CDNB (1-chloro,2,4-dinitrobenzene) and 50 mM of GSH. Optical density at 340 nm was at intervals of 1min for 5min, utilizing a Jenway Spectrophotometer-7205 UV/Vis., UK.

### 2.7 Data analysis

The data was analyzed with SPSS V.22, a statistical program. Data were analyzed for parametric test assumptions, and the normality of continuous variables was confirmed using the Shapiro–Wilk and Kolmogorov–Smirnov tests. The arcsine square root method was used to standardize the data. The mean and standard deviation of the biological, adult reproduction parameters and biochemical data were calculated using one-way ANOVA, with three replicates for each group. For the *post-hoc* analysis, Tukey’s pairwise comparison was utilized. Chi (*χ* ^2^) was used to compare the actual and expected frequencies of the sex ratio utilizing MiniTab (V. 14). A *P*-value below 0.05 indicates a significant result. In addition, the correlation coefficient relationship between enzyme activities in both female and male *S. frugiperda* after exposure to the LC_10_ and LC_50_ concentrations of chlorantraniliprole was computed, whereas the analysis became available using SigmaPlot V12.0. Thus, data visualization (V. 2022.02.4) was performed using R studio.

## 3 Results

### 3.1 Moth bioassay

The bioassay results revealed that chlorantraniliprole had potent toxicity to *S. frugiperda* moths, with an LC_50_ of 1.29 mg/L, followed by indoxacarb (13.33 mg/L), lambda-cyhalothrin (19.39 mg/L), chlorfenapyr (41.68 mg/L), and spinetoram (45.30 mg/L) (**Table 1**). Based on these results, chlorantraniliprole was selected for further sublethal and lethal effect evaluations at LC_10_ and LC_50_ concentrations.

**Table 1:** Toxicity of lambda-cyhalothrin, indoxacarb, chlorantraniliprole, chlorfenapyr, and spinetoram to *Spodoptera frugiperda* moth.

Insecticides	LC_10_ (mg/L) (95% confidence limit)	LC_50_ (mg/L) (95% confidence limit)	Slope ± SE	*χ* ^2^	*P*-value
Lambda-cyhalothrin	5.13 (0.50–9.73)	19.39 (10.63–32.99)	2.22 ± 0.69	0.32	0.84
Indoxacarb	2.16 (0.12–5.01)	13.33 (6.39–24.45)	1.62 ± 0.48	0.33	0.95
Chlorantraniliprole	0.22 (0.012–0.53)	1.29 (0.56–2.24)	1.68 ± 0.49	0.36	0.94
Chlorfenapyr	6.76 (0.38–15.66)	41.68 (19.97–76.42)	1.62 ± 0.48	0.33	0.95
Spinetoram	6.32 (0.20–15.64)	45.30 (20.80–90.25)	1.49 ± 0.47	0.90	0.82

### 3.2 Lethal and sublethal effects on offspring traits

**Figure 1:**
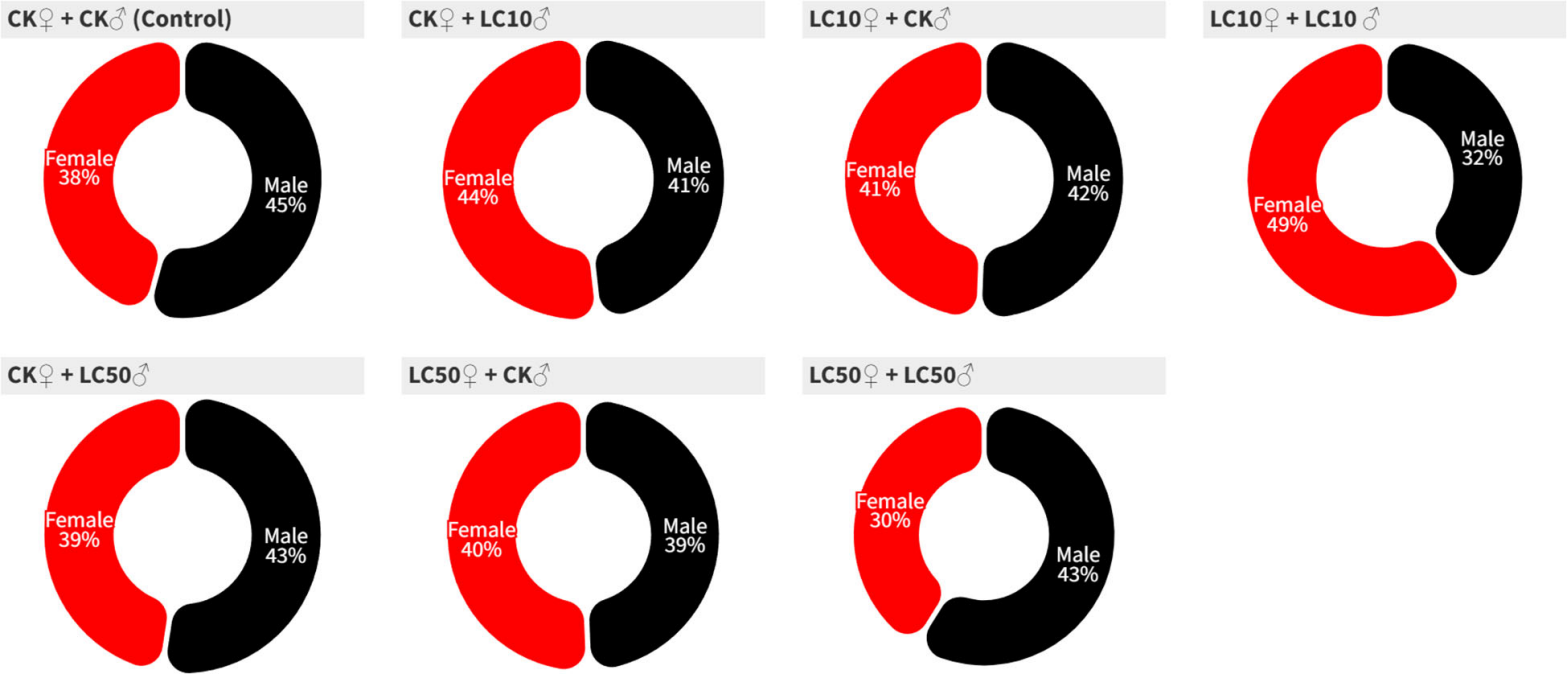
Circle chart representing the sex ratio of the emerged adults of *S. frugiperda* after treating the adults with LC_10_ and LC_50_ of chlorantraniliprole.

Exposure of parental adults to lethal (LC_50_) and sublethal (LC_10_) levels of chlorantraniliprole significantly reduced the developmental durations of both larval [F6, 598 = 8.91, *P*=0.0001] and pupal stages [F6, 555 = 8.55, *P*=0.0001] in comparison with the control (**Table 2**). However, larval mortality [F6, 14 = 0.62, *P*=0.710], pupation percentage [F6, 14 = 0.77, *P*=0.605], and adult emergence percentage [F6, 14 = 0.39, *P*=0.874] were not significantly affected. The pupal weight of both male and female moths exhibited a significant reduction in the LC_10_ groups (**Table 2**). However, chi-square (*χ*²) tests revealed no treatment-related variations in sex ratio across the experimental groups (**Table 3**, **Figure 1**).

**Table 2:** Developmental parameters (mean ± SD) of *S. frugiperda* after exposure of adults to sublethal and lethal concentrations (LC_10_ and LC_50_) of chlorantraniliprole.

Treatments	Larval duration (days)	Larval mortality, %	Pupal duration (days)	Pupation, %	Adult emergence, %	Male pupal weight (g)	Female pupal weight (g)
CK♀ + CK♂ (Control)	18.16 ± 1.01^a^	3.34 ± 3.34^a^	13.29 ± 1.58^a^	97.66 ± 2.03^a^	97.78 ± 3.85^a^	0.251 ± 0.021^ab^	0.25 ± 0.025^ab^
CK♀ + LC_10_♂	17.55 ± 1.58^b^	2.22 ± 3.85^a^	12.95 ± 1.25^ab^	96.5 ± 3.58^a^	100 ± 0^a^	0.213 ± 0.029^c^	0.208 ± 0.026^d^
LC_10_♀ + CK♂	17.38 ± 1.5^bc^	5.56 ± 6.94^a^	13.23 ± 1.36^a^	95.21 ± 4.18^a^	97.66 ± 2.03^a^	0.234 ± 0.035^b^	0.242 ± 0.031^b^
LC_10_♀ + LC_10_ ♂	17.09 ± 1.69^bc^	2.22 ± 3.85^a^	13.01 ± 1.55^ab^	94.2 ± 4.26^a^	97.7 ± 1.99^a^	0.246 ± 0.024^ab^	0.224 ± 0.027^c^
CK♀ + LC_50_♂	17.6 ± 1.13^ab^	3.34 ± 3.34^a^	12.33 ± 1.82^bc^	96.51 ± 3.45^a^	97.53 ± 4.28^a^	0.252 ± 0.026^a^	0.243 ± 0.02^b^
LC_50_♀ + CK♂	17.4 ± 1.02^bc^	3.34 ± 3.34^a^	12.18 ± 1.49^c^	93.09 ± 0.24^a^	97.62 ± 4.13^a^	0.246 ± 0.032^ab^	0.247 ± 0.024^ab^
LC_50_♀ + LC_50_♂	16.86 ± 0.75^c^	7.78 ± 5.09^a^	12.16 ± 1.56^c^	91.79 ± 7.38^a^	95.94 ± 4.17^a^	0.261 ± 0.022^a^	0.262 ± 0.024^a^
F	8.91	0.62	8.55	0.77	0.39	13.33	19.14
*P*-value	0.0001	0.710	0.0001	0.605	0.874	0.0001	0.0001

The same letter within the same column are not significantly different according to Tukey’s HSD test at 0.05 level of probability.

**Table 3:** Sex ratio (mean number) of the emerged adults of *S. frugiperda* after treating the adults with LC_10_ and LC_50_ of chlorantraniliprole.

Treatments	Male (no.)	Female (no.)	Chi-square	*P*-value
CK♀ + CK♂ (control)	45	38	0.590361	0.442
CK♀ + LC_10_♂	41	44	0.105882	0.745
LC_10_♀ + CK♂	42	41	0.012048	0.913
LC_10_♀ + LC_10_ ♂	32	49	3.5679	0.059
CK♀ + LC_50_♂	43	39	0.195122	0.659
LC_50_♀ + CK♂	39	40	0.012658	0.91
LC_50_♀ + LC_50_♂	43	30	2.31507	0.128

### 3.3 Lethal and sublethal effects on reproduction and longevity

Chlorantraniliprole exposure at both LC_10_ and LC_50_ concentrations caused a reduction in egg production per female and the hatchability percentage; however, these reductions were insignificant relative to the control [number of eggs: F6, 14 = 1.13, *P*=0.396; hatchability: F6, 14 = 1.68, *P*=0.198] (**Table 4**). A significant decline was noted in the number of hatched larvae among all treatment groups [F6, 14 = 6.34, *P*=0.002] (**Table 4**). Moreover, female longevity was significantly shortened in the LC_10_♀ + LC_10_♂ and LC_50_♀ + LC_50_♂ groups (7.48 ± 0.16 and 7.07 ± 0.56 days, respectively) relative to the control (9.01 ± 0.21 days) [*F*=4.48, *P*=0.010]. Correspondingly, the survival rates declined to 70% and 66.67% in the LC_10_ and LC_50_ groups, respectively, relative to 90% in the control (**Table 4**).

**Table 4:** Sublethal and lethal effects of chlorantraniliprole on the number of egg/female (fecundity), number of hatch, hatchability (%), survival rate, and longevity.

Treatments	Mean ± SD				
	No. of eggs/female	No. of hatch	Hatchability, %	Survival rate (%)	Longevity (days)
CK♀ + CK♂ (Control)	978.18 ± 68.93^a^	935.43 ± 94.4^a^	95.62 ± 6.47^a^	90 ± 10^a^	9.01 ± 0.21^a^
CK♀ + LC_10_♂	965.58 ± 147.66^a^	816.54 ± 46.06^ab^	85.35 ± 7.74^a^	80 ± 0^ab^	7.58 ± 0.14^ab^
LC_10_♀ + CK♂	803.88 ± 116.45^a^	652.75 ± 155.17^bc^	80.58 ± 7.86^a^	80 ± 0^ab^	7.33 ± 0.63^b^
LC_10_♀ + LC_10_ ♂	883.57 ± 42.9^a^	678.71 ± 35.94^bc^	77.06 ± 7.61^a^	70 ± 0^b^	7.48 ± 0.16^b^
CK♀ + LC_50_♂	913.9 ± 170.11^a^	733.05 ± 39.5^abc^	81.45 ± 10.15^a^	70 ± 0^b^	7.76 ± 0.92^ab^
LC_50_♀ + CK♂	798.87 ± 173.77^a^	615 ± 117.95^bc^	77.63 ± 10.03^a^	66.67 ± 5.77^b^	7.51 ± 0.45^b^
LC_50_♀ + LC_50_♂	768.44 ± 176.83^a^	561.5 ± 47.27^c^	75.06 ± 13.41^a^	66.67 ± 5.77^b^	7.07 ± 0.56^b^
F	1.13	6.34	1.68	9.73	4.48
*P*-value	0.396	0.002	0.198	0.0001	0.010

The same letter within the same column are not significantly different according to Tukey’s HSD test at 0.05 level of probability.

### 3.4 Enzyme activity

Following exposure to LC_10_ and LC_50_ concentrations, no significant differences were detected in the activities of cytochrome P450, α-esterase, and GST in female moths compared to the control [α-esterase: F2, 6 = 0.12, *P*=0.886; P450: F2, 6 = 1.38, *P*=0.321; GST: F2, 6 = 0.95, *P*=0.437] (**Table 5**). In male moths, GST activity significantly increased at LC_50_ [F2, 6 = 8.52, *P*=0.018], while α-esterase and cytochrome P450 activities remained statistically unchanged across treatments (**Table 5**).

**Table 5:** Activities (mean ± SD) of detoxification enzymes in *S. frugiperda* adults following exposure to sublethal and lethal (LC_10_ and LC_50_) concentrations of chlorantraniliprole.

Treatments		α-esterase (mmol/mg of protein)	Cytochrome P450 (nmol/min/mg of protein)	GST (µmol/min/mg of protein)
Female	Cont.	0.022 ± 0.004^a^	24.75 ± 4.29^a^	2.31 ± 0.56^a^
	LC_10_	0.023 ± 0.005^a^	31.15 ± 4.85^a^	2.49 ± 0.26^a^
	LC_50_	0.023 ± 0.002^a^	29.06 ± 5.25^a^	2.87 ± 0.61^a^
*F*		0.12	1.38	0.95
*P*-value		0.886	0.321	0.437
Male	Cont.	0.021 ± 0.005^a^	22.53 ± 1.56^a^	2.83 ± 0.38^b^
	LC_10_	0.019 ± 0.002^a^	28.84 ± 5.94^a^	2.98 ± 0.35^b^
	LC_50_	0.02 ± 0.004^a^	32.3 ± 9.27^a^	3.89 ± 0.28^a^
*F*		0.22	1.79	8.52
*P*-value		0.810	0.246	0.018

The same letter within the same column are not significantly different according to Tukey’s HSD test at 0.05 level of probability.

### 3.5 Correlation between enzyme activity in male and female individuals of *S. frugiperda*

**Figure 2:**
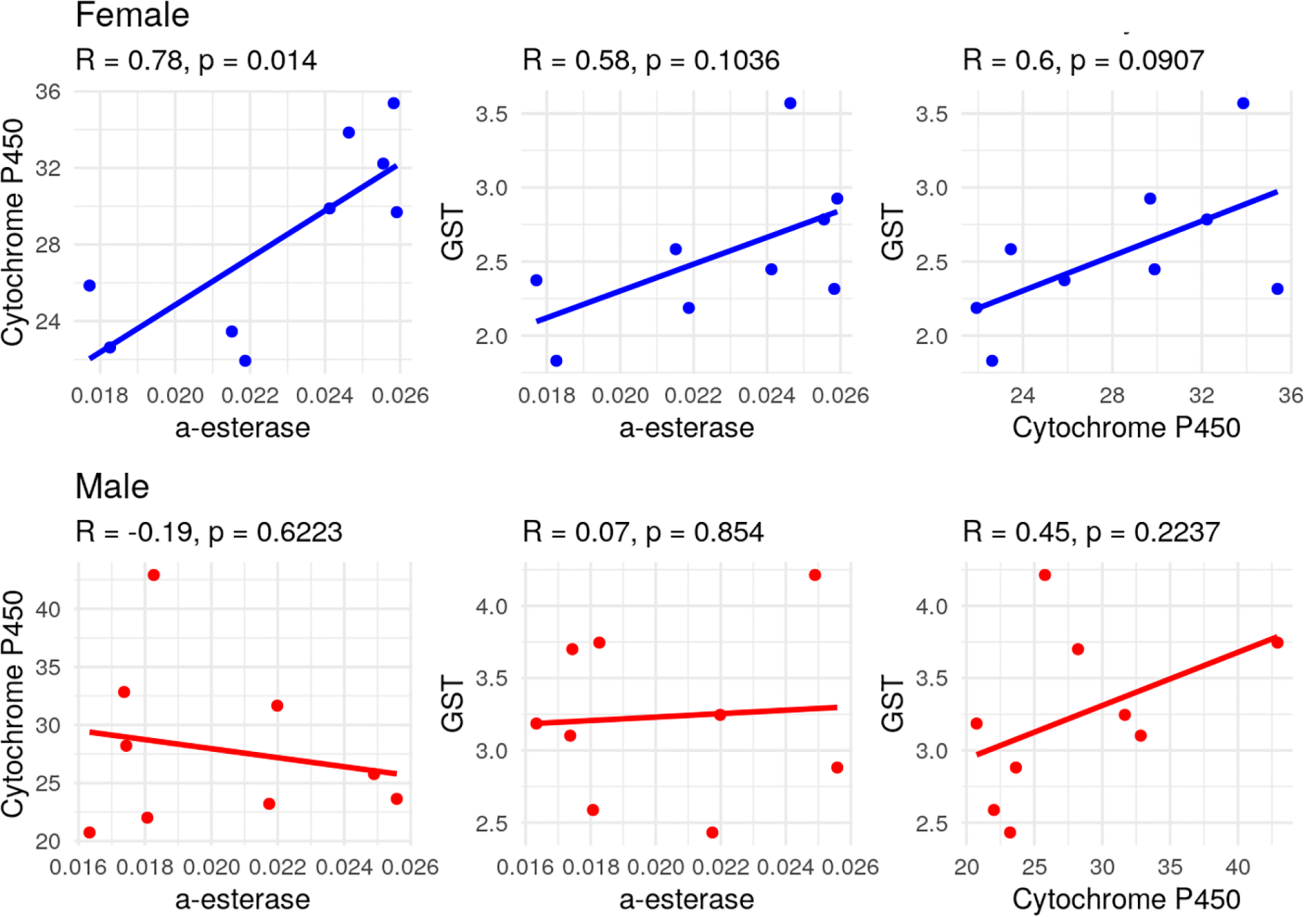
Matrix plot representing the correlation relationship between enzymatic activity in both female and male individuals of *S. frugiperda* after having been exposed to the LC_10_ and LC_50_ concentrations of chlorantraniliprole.

A strong positive correlation was observed between α-esterase and cytochrome P450 activities in female adults (*R*=0.78, *P*=0.014), indicating that higher α-esterase levels were associated with increased cytochrome P450 activity (**Figure 2**). Moderate but non-significant positive correlations were also found between α-esterase and GST (*R*=0.58, *P*=0.103) and between cytochrome P450 and GST (*R*=0.60, *P*=0.090). In male adults, the correlation analysis revealed weak negative to moderate positive associations between enzyme activities, but none was statistically significant (**Figure 2**).

## 4 Discussion

Insect pests such as *S. frugiperda* impose significant threats to global agriculture, resulting in considerable economic losses and environmental costs (Gul et al., 2023). Although chemical insecticides remain widely employed for pest control (Moustafa et al., 2022, 2023), their effectiveness is often compromised by environmental degradation factors, including ultraviolet (UV) radiation, sunlight, and photolysis (Moustafa et al., 2018). Consequently, insects are frequently subjected to sublethal concentrations, which can significantly alter biological traits and enzymatic activities (Kandil et al., 2020).

Our research assessed the lethal and sublethal impacts of selected insecticides, with a focus on chlorantraniliprole, on *S. frugiperda* adults and their offspring. Within the studied compounds, chlorantraniliprole revealed the highest toxicity (LC_50_ = 1.29 mg/L). Generally, chlorantraniliprole has the potential to become one of the most promising compounds in pest management (Moustafa et al., 2023), including *S. frugiperda* (Zhang et al., 2020; Akhtar et al., 2022; Chen et al., 2023).

Exposure of parental adults to lethal (LC_50_) and sublethal (LC_10_) concentrations of chlorantraniliprole significantly reduced larval and pupal developmental durations, reduced pupal weights, and shortened adult longevity in their progeny, although larval mortality, pupation rate, and emergence rate were not significantly affected. Exposure to chlorantraniliprole seems to reduce the ability of *S. frugiperda* to reproduce and maintain a stable population (Akhtar et al., 2022; Abbas et al., 2025).

Previous findings indicated that chlorantraniliprole, a chemical compound related to anthranilic diamides, has been found to negatively influence the population of lepidopteran larvae (Lai and Su, 2011; Han et al., 2012; Guo et al., 2013; Zhang et al., 2013; Carneiro et al., 2016; Lutz et al., 2018). Importantly, chlorantraniliprole is considered highly selective with low toxicity toward natural enemies, making it an ideal candidate for integration into environmentally sustainable pest management programs (Brugger et al., 2010). Additional comparative studies reinforce the value of chlorantraniliprole. Zhang et al. (2022) demonstrated its high activity against *Agrotis ipsilon* (Hufnagel) and *Agrotis segetum* (Denis & Schiffermuller) adults, significantly reducing fecundity, egg hatchability, flight distance, and population growth. Similarly, Ismail (2020) found that chlorantraniliprole at low concentrations induced high mortality rates in *A. ipsilon* adults, with LC_50_ values varying across species. Our findings are consistent with these studies, confirming chlorantraniliprole’s high potency against adult moths, although species-specific sensitivity variations exist. Moreover, Liu et al. (2017) reported that chlorantraniliprole demonstrated greater toxicity against lepidopteran moths (*A. ipsilon*, *Helicoverpa armigera* Hubner, and *Spodoptera litura* Fabricius) and rapid efficacy compared to other insecticides such as methomyl, spinetoram, and emamectin benzoate, making it a preferable candidate for attract-and-kill systems. Although emamectin benzoate is toxic, its effects act too slowly to achieve fast control in strategies needed in adult-targeting strategies (Liu et al., 2017). Quickly rendering the insect incapable of movement reduces the chance of egg laying after being exposed to the insecticide.

Since chlorantraniliprole is both highly effective and environmentally safe, it can be safely used together with BioAttract, helping to attract and eliminate insects (attract-and-kill strategies).

As an obligate migratory pest, *S. frugiperda*’s high fecundity and strong flight capabilities contribute to its rapid regional spread and frequent outbreaks (Zhang et al., 2022). Trying to kill adult moths that feed on certain attractants can reduce their number and prevent them from migrating. Our findings indicate that controlling pests at the adult stage is very important (Zhang et al., 2022).

The larval stages of insects are found to be more sensitive to insecticides than the adult life stage (Xie et al., 2010; Kong et al., 2021). This observation aligns with findings that detoxification enzyme activity (e.g., CarE, GST, and MFOs) varies between larvae and adults (Sívori et al., 1997; Qiu and Zhang, 2001; Wang et al., 2008; Ou et al., 2012). Numerous studies have examined the functions of α-esterase, cytochrome P450, and GST, especially in relation to the insects’ exposure to insecticides (Moustafa et al., 2025). Because of their sensitivity in signaling chemical exposure, these detoxifying enzymes are powerful biological markers (Moustafa et al., 2023). Nevertheless, it has been noted that different insects’ responses to different chemicals exhibit varying degrees of activation and inhibition in their activity following insecticide exposure (Su and Xia, 2020). In our study, enzymatic assays indicated a minimal induction of detoxification pathways following chlorantraniliprole exposure, suggesting a lower risk of rapid resistance development at the adult stage.

The integration of chlorantraniliprole into attract-and-kill schemes, rather than conventional field sprays, could help delay resistance evolution and extend the effective lifespan of this active ingredient (Zhang et al., 2020).

## 5 Conclusions

Our study confirmed that chlorantraniliprole exerts significant lethal and sublethal impacts on *Spodoptera frugiperda* adults and their progeny. Exposure to sublethal concentrations accelerated larval and pupal development, decreased pupal weights, shortened adult longevity, and modestly impaired reproductive output, highlighting its potential to disrupt pest population dynamics even when direct mortality is limited. The minimal induction of detoxification enzyme activities in treated adults suggests a lower risk of rapid resistance development compared to other chemical classes. Furthermore, the sublethal impacts of chlorantraniliprole, comparable to those reported for the tested insecticides, reinforce its suitability for integration into attract-and-kill strategies targeting adult moths before migration and reproduction occur. Given its high efficacy, low non-target toxicity, and ingestion-based action, chlorantraniliprole represents a promising tool for the sustainable management of migratory lepidopteran pests such as *S. frugiperda*.

## Data Availability

The original contributions presented in the study are included in the article/**Supplementary Material**. Further inquiries can be directed to the corresponding authors.
