# Supplementary material for: Lethal and sublethal effects of chemical and bio-insecticides on Spodoptera frugiperda adults: new perspectives for “attract-and-kill” control strategies
Source: Front Plant Sci. 2025 Oct 15;16:1694032. doi: 10.3389/fpls.2025.1694032 (PMC12568522; doi:10.3389/fpls.2025.1694032)

Table S1. Serial concentrations used in bioassay experiments

Insecticides	Concentrations
Lambda-cyhalothrin	60, 30, 15, 7.5, 3.75
Indoxacarb	64, 32, 16, 8, 4
Chlorantraniliprole	8, 4, 2, 1, 0.5
Chlorfenapyr	200, 100, 50, 25, 12.5
Spinetoram	200, 100, 50, 25, 12.5

Fig. S1. Bioassay and mating experimental designs

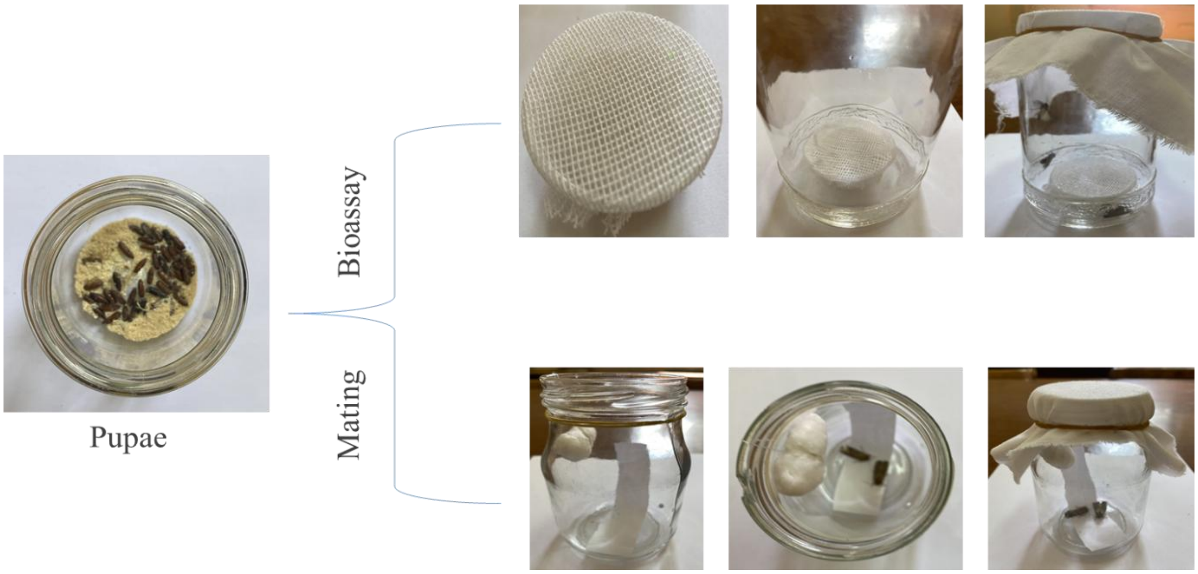

Supplement: Supplementary file 1 [file DataSheet1.pdf]
